# A Personalized FSH Dosing Strategy for Women with Polycystic Ovary Syndrome Undergoing GnRH Antagonist Protocols

**DOI:** 10.3390/biomedicines14040769

**Published:** 2026-03-28

**Authors:** Yixin Chen, Turui Yang, Zicong Luo, Lu Luo, Ziqing Zhang, Yanwen Xu, Minghui Chen

**Affiliations:** 1 Reproductive Medicine Center, The First Affiliated Hospital of Sun Yat-sen University, Guangzhou 510080, China; chenyx825@mail.sysu.edu.cn (Y.C.);; 2 Guangdong Provincial Key Laboratory of Reproductive Medicine, Guangzhou 510080, China; 3 Guangdong Provincial Clinical Research Center for Obstetrical and Gynecological Diseases, Guangzhou 510080, China

**Keywords:** polycystic ovary syndrome, FSH dosing, precision medicine, controlled ovarian stimulation, GnRH antagonist, clinical decision support

## Abstract

**Background:** Polycystic ovary syndrome (PCOS) is characterized by substantial inter-individual variability in ovarian sensitivity to recombinant follicle-stimulating hormone (rFSH) during controlled ovarian stimulation (COS). Clinically applicable tools for personalized dosing in this population remain limited. **Methods:** This retrospective single-center study (2013–2024) analyzed 369 PCOS patients undergoing GnRH antagonist protocols who achieved optimal ovarian responses (10–20 oocytes with at least 40% of follicles ≥ 16 mm in diameter on trigger day). The final retrospective dataset was randomly split into modeling (*n* = 258) and validation (*n* = 111) groups. A multivariate linear regression model incorporating age, BMI, basal FSH, basal LH, AMH, and AFC was developed to estimate the average daily rFSH dose. Model performance was evaluated using correlation analysis, prediction error metrics, and calibration assessment. **Results:** Age, BMI, and basal FSH were positively associated with average daily rFSH dose, whereas basal LH, AMH, and AFC were negatively associated. The model explained 40.4% of the variability in average daily rFSH dose. In the modeling cohort, 77.9% of estimated doses fell within ±20% of the observed values, with a moderate correlation between predicted and observed doses (*ρ* = 0.646). In the validation cohort, 67.6% of estimates met the predefined accuracy threshold (*ρ* = 0.676). Calibration analyses demonstrated robust agreement between predicted and observed doses. **Conclusions:** By integrating endocrine markers, ovarian reserve indicators, and clinical characteristics, this study provides a practical example of personalized medicine in COS in women with PCOS. The internally validated approach may support individualized rFSH dosing during COS and serve as a basis for future development of decision support tools in this specific population.

## 1. Introduction

Polycystic ovary syndrome (PCOS) is a prevalent and complex endocrine disorder affecting approximately 5% to 10% of women of reproductive age [1]. It is characterized by heterogeneous clinical and endocrine manifestations, including hyperandrogenism, menstrual irregularity and polycystic ovarian morphology [2]. PCOS is associated with various reproductive impairments, including anovulatory infertility and pregnancy complications [3]. Importantly, pronounced inter-individual variability in ovarian reserve, hormonal milieu, and sensitivity to recombinant follicle-stimulating hormone (rFSH) makes PCOS a paradigmatic condition of endocrine heterogeneity.

During assisted reproductive technology (ART), women with PCOS frequently exhibit heightened sensitivity to rFSH stimulation. While excessive ovarian sensitivity increases the risk of ovarian hyperstimulation syndrome (OHSS), an iatrogenic and potentially life-threatening complication [4], insufficient rFSH exposure may result in inadequate follicular recruitment and suboptimal ovarian response [5]. These opposing risks highlight the intrinsic limitations of conventional “one-size-fits-all” or empirically adjusted dosing strategies, which may compromise treatment safety, efficiency, and resource utilization in this population.

In clinical practice, physicians typically determine the initial dose of rFSH by relying on their clinical experience, considering factors such as the patient’s antral follicle count (AFC), age, body mass index (BMI), and responses to prior ovarian stimulation cycles. To improve dose individualization, various studies have employed clinical characteristics and biological indicators to establish predictive models for determining the initial rFSH dose in patients. These models have explored correlations between factors such as age, BMI, AFC, and anti-Müllerian hormone (AMH) with the number of retrieved oocytes, subsequently formulating linear regression models and visualizing these findings through algorithms or nomograms. However, these studies either excluded women with PCOS or failed to differentiate between individuals with PCOS and those without [6,7,8,9,10]. Given the distinct biological characteristics and exaggerated ovarian sensitivity observed in PCOS, direct extrapolation of these models to this subgroup remains clinically questionable.

From a precision medicine perspective, individualized rFSH dosing requires not only consideration of baseline patient characteristics but also an assessment of treatment intensity throughout the stimulation course. Most existing dosing models focus primarily on initial rFSH prescriptions, which may not adequately capture subsequent dose adjustments made in response to follicular development and hormonal dynamics during controlled ovarian stimulation (COS). In this context, the average daily rFSH dose administered throughout the stimulation period may therefore provide a more comprehensive reflection of personalized ovarian stimulation management during COS than the starting dose alone.

Accordingly, we aimed to develop a clinically applicable algorithm to estimate the average daily rFSH dose required for women with PCOS undergoing gonadotropin-releasing hormone (GnRH) antagonist protocols. By integrating various clinical characteristics and biological markers, this model generates individualized dose estimates to support rFSH dosing decisions during COS. Unlike previous studies that focused mainly on the initial FSH dose or mixed infertility populations, the present study specifically addressed the average daily rFSH dose in women with PCOS undergoing GnRH antagonist cycles. Its intended role is to help improve dose selection, increase the likelihood of achieving an optimal ovarian response, and reduce avoidable empirical variation in routine practice. Taken together, this work illustrates a practical application of precision medicine in COS in women with PCOS and provides a methodological basis for future development of clinical decision support tools in this clinical setting.

## 2. Material and Methods

### 2.1. Ethics Statement

This study was approved by the Ethics Committee of the First Affiliated Hospital of Sun Yat-sen University (Approval No. 2023(029); Date: 24 May 2023). All procedures were conducted in accordance with relevant guidelines and regulations.

### 2.2. Population

This retrospective, single-center study included patients aged 20–40 years diagnosed with PCOS who underwent IVF, ICSI, or preimplantation genetic testing (PGT) using a GnRH antagonist protocol at the First Affiliated Hospital of Sun Yat-sen University. The study period spanned from January 2013 to December 2024. PCOS diagnosis was made in accordance with the Rotterdam criteria. Eligible women/cycles were included consecutively during the study period after application of the predefined inclusion and exclusion criteria. The exclusion criteria were as follows: endocrine diseases such as the pituitary, thyroid, adrenal, and pancreas disorders; history of ovarian surgery; endometriosis; ovarian tumors; congenital adrenal hyperplasia; Cushing’s syndrome; a history of chemotherapy and/or radiotherapy; obesity (BMI ≥ 30 kg/m^2^); and cycles complicated by moderate or severe OHSS.

For model development, the study cohort was further restricted to cycles with an optimal ovarian response, defined as 10–20 retrieved oocytes and a proportion of bilateral follicles ≥ 16 mm on the trigger day ≥ 0.4. This restriction was intended to improve cohort homogeneity for model development. The final retrospective dataset was randomly split into the modeling and validation groups in a 7:3 ratio using the caret package in R software for internal validation of the predictive algorithm.

### 2.3. Fixed Day 5/6 GnRH Antagonist Protocol

COS was conducted using a fixed day 5/6 GnRH antagonist protocol [11,12]. Ovarian stimulation was initiated on menstrual cycle days 2–4 with recombinant follicle-stimulating hormone (rFSH; Merck Serono, Darmstadt, Germany). The initial rFSH dose, ranging from 150 to 300 IU, was individualized according to patient age, BMI, and ovarian reserve. During stimulation, rFSH dose adjustments were made by the physicians based on baseline patient characteristics and routine clinical monitoring. A GnRH antagonist—cetrorelix acetate (Merck Serono, Darmstadt, Germany) or ganirelix acetate (Organon, Oss, The Netherlands)—was administered at a daily dose of 250 μg starting on stimulation day 5 or 6 irrespective of follicular development criteria.

Triggering was performed when at least three follicles reached a mean diameter of ≥18 mm. For patients at high risk of OHSS, a dual trigger was applied consisting of human chorionic gonadotropin (hCG; Livzon Pharmaceutical Group, Zhuhai, China) at a dose of 2000 IU combined with a GnRH agonist, triptorelin acetate (Ferring Pharmaceuticals, Saint-Prex, Switzerland), at a dose of 0.2 mg. Patients at standard risk received recombinant hCG (choriogonadotropin alfa; Merck Serono, Darmstadt, Germany) alone at a dose of 250 μg. Oocyte retrieval was scheduled 34–36 h after trigger administration.

### 2.4. Hormone Assay and AFC Measurement

Prior to ovarian stimulation, basal serum levels of AMH, FSH, luteinizing hormone (LH), estradiol (E2), progesterone (P), and testosterone (T) were assessed from blood samples collected on menstrual cycle day 2. Analysis utilized the Beckman Coulter Access Immunoassay System (Beckman Coulter, Brea, CA, USA) based on chemiluminescence principles. Assays were performed strictly adhering to manufacturer protocols, with all reported results falling within validated quality control ranges. Ovarian follicles measuring 2–10 mm in diameter were counted bilaterally via transvaginal ultrasound on the same day as blood sampling. The sum of these follicles from both ovaries represented the AFC.

### 2.5. Variables Selection

The association between average daily rFSH dose and variables including age, BMI, AMH, AFC, basal FSH, and basal LH was analyzed using univariate linear regression. Variables that demonstrated statistical significance in the univariate analysis were included in the construction of the algorithm.

### 2.6. Outcome Measurement

The primary outcome was the average daily rFSH dose during stimulation up to the trigger day (IU/day), which served as the prediction target for model development. It was calculated as follows:

average daily rFSH dose (IU/day) = cumulative rFSH dose administered from stimulation start to trigger day (IU)/duration of rFSH administration (days)

Trigger medications, including hCG and/or GnRH agonist, were not included in the rFSH dose calculation.

### 2.7. Statistical Analysis

#### 2.7.1. Descriptive Analysis

Continuous variables were tested for normality using the Shapiro–Wilk test. Normally distributed data were presented as the mean ± standard deviation (SD) and analyzed via independent t-tests, whereas non-normally distributed data were expressed as the median (interquartile range, IQR) and analyzed via Mann–Whitney U tests. Categorical variables were expressed as percentages (%) and compared using Pearson’s chi-square test. Statistical analyses were performed using SPSS (version 26.0; IBM Corp., Armonk, NY, USA).

#### 2.7.2. Univariate and Multivariate Linear Regression

Univariate linear regression was performed to assess the associations between average daily rFSH dose and clinical variables including age, BMI, basal FSH, basal LH, AMH, and AFC. Variables with *p* < 0.05 were included in a multivariate linear regression to identify independent predictors. Results were reported as the unstandardized regression coefficient (B) and its 95% confidence interval (95% CI). Model performance was evaluated using the adjusted *R*^2^.

#### 2.7.3. Internal Validation of the Predictive Algorithm

The predictive algorithm was internally validated using randomly split modeling and validation subsets derived from the same retrospective institutional cohort. In each subset, model performance was evaluated by comparing the predicted and observed average daily rFSH doses. Agreement between the two was quantified using Spearman’s correlation coefficient (*ρ*), interpreted as negligible (0.0–0.3), low (0.3–0.5), moderate (0.5–0.7), high (0.7–0.9), and very high (0.9–1.0) [13].

Prediction error was assessed using the mean absolute error (MAE), reflecting the magnitude of deviation from observed values, and the root mean squared error (RMSE), reflecting dispersion in the original units. Clinical applicability was examined by calculating the proportion of predictions falling within 20% of the actual dose (deviation ≤ 20%), which was predefined as the acceptable clinical threshold.

Calibration assessment was performed separately for the modeling and validation groups. Observed average daily rFSH doses were regressed on predicted values to obtain the calibration slope and intercept, indicating systematic agreement or bias. The coefficient of determination (*R*^2^) and adjusted *R*^2^ quantified the overall goodness of fit. Calibration plots were generated in R software (version 4.3.2; R Foundation for Statistical Computing, Vienna, Austria), with the 45° dashed line representing perfect calibration.

## 3. Results

### 3.1. Baseline Characteristics and Treatment Characteristics

A total of 369 infertile women with PCOS who underwent IVF/ICSI/PGT between January 2013 and December 2024 were analyzed. Within this cohort, 258 patients constituted the modeling group, and 111 formed the validation group. Baseline characteristics and outcomes of treatment were comparable between the two groups (Table 1).

### 3.2. Factors Associated with Average Daily rFSH Dose

**Figure 1:**
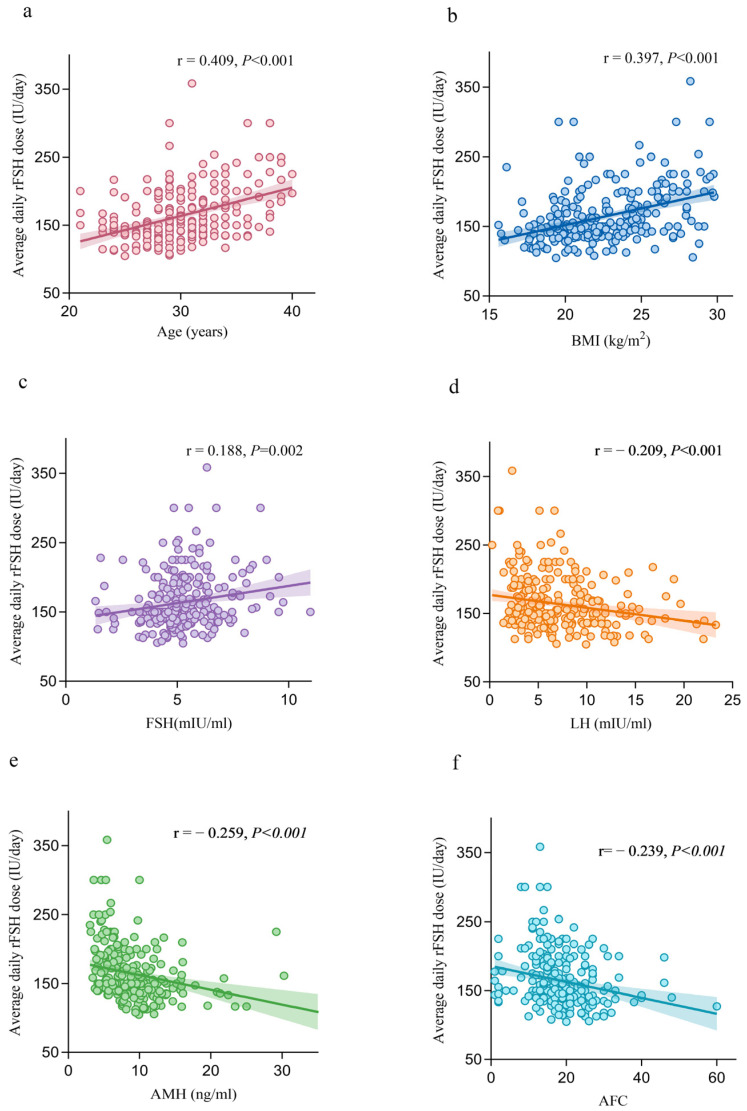
Fitted linear relationships of average daily recombinant follicle-stimulating hormone (rFSH) dose to clinical predictors. Scatter plots (**a**–**f**) depicting the linear regression fits between average daily rFSH dose (IU/day) and key clinical predictors: (**a**) Age (years), (**b**) Body Mass Index (BMI, kg/m^2^), (**c**) Basal FSH (mIU/mL), (**d**) Basal luteinizing hormone (LH, mIU/mL), (**e**) Anti-Müllerian hormone (AMH, ng/mL), and (**f**) Antral follicle count (AFC, n). Shaded bands indicate the 95% confidence intervals (95% CI) around the regression line. Pearson’s correlation coefficient (r) and associated *p*-value (*p* < 0.01 for all predictors) are displayed in each subplot.

The univariate linear regression analysis demonstrated significant associations between the average daily rFSH dose and several clinical predictors. Specifically, positive associations were observed with age (r = 0.409, *p* < 0.001), BMI (r = 0.397, *p* < 0.001) and basal FSH (r = 0.188, *p* = 0.002). Conversely, negative associations were identified with basal LH (r = −0.209, *p* = 0.0007), AMH (r = −0.259, *p* < 0.001) and AFC (r = −0.239, *p* < 0.001) (Figure 1).

These six variables were subsequently entered into a multivariate linear regression model, all of which remained statistically significant predictors of average daily rFSH dose (Table 2). Based on the multivariate analysis, the predictive algorithm for estimating the average daily rFSH dose was formulated as follows:

Average daily rFSH dose=−28.281+3.120×Age+4.260×BMI+6.790×Basal FSH−1.806×Basal LH−0.881×AMH−0.706×AFC

where Age (years), BMI (kg/m^2^), Basal FSH (mIU/mL), Basal LH (mIU/mL), AFC (n), and AMH (ng/mL) are the respective variables considered in the model.

### 3.3. Calibration and Validation of the Algorithm

Model performance metrics for the modeling and validation groups are summarized in Table 3.

In the modeling group, predictions showed excellent concordance with observed average daily rFSH doses (*p* = 0.986) and moderate correlation (*ρ* = 0.646), with clinically acceptable accuracy (MAE = 21.82 IU, RMSE = 29.75 IU) and 77.91% of predictions within ≤20% deviation. The calibration slope (1.022) and intercept (−3.646) were close to ideal, and the adjusted *R*^2^ was 0.402, indicating good internal fit.

In the validation group, the paired *t*-test showed no significant difference between predicted and observed values (*p* = 0.540). In addition, correlation strength improved (*ρ* = 0.676) and error metrics remained comparable (MAE = 23.87 IU, RMSE = 31.89 IU), with 67.57% of predictions within 20% deviation. The calibration slope (1.286) and intercept (−49.364) suggested mild overestimation at higher predicted doses, yet the adjusted *R*^2^ remained satisfactory at 0.479.

**Figure 2:**
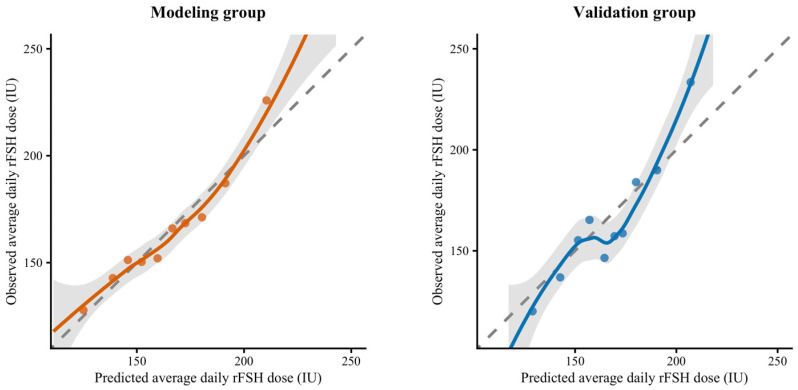
Calibration of the predictive algorithm for average daily rFSH dose in the modeling and validation cohorts.

Calibration plots (Figure 2) show that predicted and observed doses aligned closely along the 45° reference line in both groups, confirming robust internal validation and acceptable generalizability within the single-center cohort.

Calibration plots illustrate the agreement between predicted and observed average daily rFSH doses in the modeling (left) and validation (right) groups.

The solid colored lines represent locally estimated scatterplot smoothing (LOESS) fits of the observed values, and the shaded bands denote the corresponding 95% confidence intervals. The gray dashed line indicates perfect calibration (observed = predicted). In the modeling group, the calibration slope and intercept were close to ideal, demonstrating satisfactory agreement between predicted and observed doses. In the validation group, mild overestimation was observed at higher predicted values, but overall calibration remained acceptable.

## 4. Discussion

This study developed a clinically applicable algorithm for estimating the average daily rFSH dose in women with PCOS undergoing GnRH antagonist protocols. By integrating routinely available clinical parameters, the model addresses an important clinical challenge in COS for women with PCOS: achieving individualized rFSH dose selection in a population characterized by marked heterogeneity in ovarian response.

Appropriate rFSH dosing promotes follicular growth and synchronizes oocyte maturation at retrieval. Several predictive models for individualized FSH dosing have been reported. Popovic-Todorovic et al. developed a nomogram incorporating ovarian stromal blood flow and AFC [6], Howles et al. proposed the CONSORT formula based on age, BMI, basal FSH, and AFC [7], and La Marca et al. constructed a nomogram using age, AMH, and basal FSH in agonist protocols [8]. More recently, Li et al. reported the first nomogram for antagonist cycles using BMI, AMH, and AFC [10]. While these studies provide valuable references for FSH dosing strategies, they either excluded PCOS patients or did not differentiate PCOS from other populations. This represents an important limitation, as PCOS patients exhibit stronger ovarian reserve and heightened sensitivity to exogenous rFSH. Recent evidence has demonstrated significant differences in total rFSH requirements and oocyte yield between PCOS and non-PCOS infertile populations [14]. Consequently, determining an appropriate rFSH dose for PCOS patients remains a substantial clinical challenge.

In this retrospective cohort study, multivariate linear regression confirmed that age, BMI, AMH, AFC, basal FSH and basal LH levels were independent predictors of average daily rFSH dose. To our knowledge, this is the first algorithm specifically developed to estimate the average daily rFSH dose in women with PCOS undergoing a GnRH antagonist protocol.

To construct a training set with high clinical relevance, we defined the optimal oocyte yield as 10–20 oocytes, consistent with previous studies showing that this range is associated with higher cumulative live birth rates while maintaining OHSS risk at an acceptable level [15,16,17,18]. Additionally, we included patients in the research only if the proportion of follicles exceeding 16 mm in diameter on trigger day was greater than 0.4. COS may result in the developmental heterogeneity of follicles, wherein the maturity and quality of the oocytes can vary greatly. Studies have consistently demonstrated that follicular size is correlated with oocyte developmental competence, fertilization potential, and subsequent embryo quality [19,20]. Specifically, follicles larger than 16 mm on trigger day are significantly more likely to yield mature oocytes and achieve successful fertilization [21,22,23]. This selection criterion ensured that the algorithm was developed using cycles with both effective stimulation and appropriate follicular maturation. This design was intentional, as the aim of the model was to learn the stimulation-phase rFSH dose pattern associated with clinically favorable stimulation performance.

Unlike prior investigations that analyzed only initial rFSH dose, we used average daily rFSH dose as the outcome. This approach reflects real-world COS management, where physicians set the initial dose empirically and adjust it based on follicular growth and hormone monitoring. Consequently, average daily rFSH dose provides a more comprehensive reflection of stimulation intensity throughout the ovarian stimulation cycle than the starting dose alone. Although it is not a final reproductive outcome, it is a clinically meaningful stimulation-phase management target because dose selection directly influences ovarian response and subsequent COS outcomes, including oocyte yield. In women with PCOS, this may have particular clinical relevance, as marked inter-individual variability in ovarian sensitivity makes dose selection especially challenging during COS. By estimating the average daily rFSH dose, the present model may provide a practical reference for both initial dose selection and subsequent dose adjustments during COS in this population.

Notably, basal LH emerged as a negative predictor of average daily rFSH dose, distinguishing this model from prior algorithms developed for general infertile populations. This finding is consistent with the endocrine characteristics of PCOS and suggests that LH may be an informative marker in individualized rFSH dose selection for this population.

This study has several strengths. It focused exclusively on women with PCOS undergoing GnRH antagonist protocols, a population in whom ovarian sensitivity is highly heterogeneous. The model development cohort was further restricted to cycles with an optimal ovarian response and adequate follicular maturation, thereby improving the clinical relevance of the training set. In addition, unlike previous studies that concentrated mainly on the initial FSH dose, we used the average daily rFSH dose as the outcome, which may reflect stimulation intensity across the entire COS course more comprehensively. Finally, the model was developed using routinely available variables and underwent structured internal validation, supporting its potential practical applicability. Integration of such an algorithm into hospital workflow systems could potentially allow clinicians to dynamically generate average daily rFSH dose estimates based on real-time patient data, thereby facilitating more individualized and effective COS management in women with PCOS.

Limitations include the retrospective single-center design, which may introduce selection bias and limit generalizability. In addition, the model development cohort was intentionally restricted to a selected subgroup of women with PCOS, excluding women with obesity and including women with cycles with an optimal ovarian response. Although these restrictions may have improved cohort homogeneity and strengthened the clinical relevance of the training set for dose selection, they also limit the generalizability of the algorithm. This is important because BMI was an independent predictor of the average daily rFSH dose, and obesity is clinically relevant to ovarian stimulation response, ART outcomes, and treatment safety. Accordingly, the present model should be interpreted with caution outside the non-obese PCOS population and before application to women with poor response, failed COS, or adverse stimulation outcomes. The modest reduction in accuracy in the validation cohort also suggests possible overfitting, highlighting the need for evaluation in larger and more diverse populations. Future multicenter, prospective studies are needed to validate this algorithm in diverse clinical settings and to assess its impact on ART outcomes. Accurate follicle enumeration in women with PCOS also remains technically challenging, particularly because of the high number of follicles and the difficulty in counting small antral follicles. Emerging technological advances such as three-dimensional ultrasound may help enhance predictive performance in PCOS populations [24].

## 5. Conclusions

Overall, this study demonstrates that average daily rFSH dose in women with PCOS undergoing GnRH antagonist COS can be reasonably estimated using routinely available clinical and hormonal parameters. The proposed algorithm highlights the potential role of data-driven approaches in supporting individualized rFSH dose selection in daily clinical practice. Further multicenter, prospective studies are needed to confirm its generalizability and to clarify its clinical utility in broader ART settings.

## Figures and Tables

**Table 1:** Comparison of baseline characteristics and treatment outcomes between modeling group and validation group.

Variables	Modeling Group (*n* = 258)	Validation Group (*n* = 111)	*p* ^a^
Female age (years)	30.00 (28.00, 33.00)	30.00 (28.00, 32.00)	0.326
Male age (years)	32.00 (30.00, 35.00)	32.00 (29.00, 35.00)	0.492
ART methods, n (%)			0.486
IVF	159 (61.63)	64 (57.66)	
ICSI	55 (21.32)	30 (27.03)	
PGT	44 (17.06)	17 (15.32)	
Duration of infertility (years)	3.00 (2.00, 5.00)	4.00 (2.00, 5.00)	0.069
BMI (kg/m^2^)	22.02 (20.10, 24.80)	22.72 (20.37, 25.12)	0.466
AFC (n)	18.00 (14.00, 23.00)	18.00 (15.00, 23.00)	0.613
Basal FSH (mIU/mL)	5.12 (4.47, 6.01)	5.00 (4.13, 5.92)	0.263
Basal LH (mIU/mL)	5.78 (3.50, 9.04)	4.99 (3.84, 8.05)	0.431
Basal E2 (pg/mL)	24.00 (13.47, 38.75)	21.88 (13.07, 37.84)	0.604
Basal T(ng/mL)	0.44 (0.34, 0.56)	0.42 (0.33, 0.55)	0.278
AMH (ng/mL)	8.25 (6.08, 10.65)	8.17 (5.78, 10.96)	0.056
Cumulative rFSH dose (IU)	1350.00 (1125.00, 1725.00)	1350.00 (1125.00, 1800.00)	0.945
Initial rFSH dose (IU)	150.00 (150.00, 187.50)	150.00 (150.00, 175.00)	0.328
Duration of stimulation (days)	9.00 (8.00, 10.00)	9.00 (8.00, 10.00)	0.750
Average daily rFSH dose (IU/day)	150.00 (139.29, 185.16)	150.00 (137.50, 185.94)	0.867
Number of oocytes retrieved (n)	15.00 (13.00, 18.00)	16.00 (13.00, 18.00)	0.107
Number of mature oocytes (n)	13.00 (11.00, 16.00)	14.00 (11.00, 16.00)	0.169
Number of 2PN oocytes (n)	11.00 (9.00, 14.00)	12.00 (9.50, 14.00)	0.245
Number of utilizable embryos on Day 3 * (n)	4.00 (2.00, 6.00)	4.00 (2.00, 7.00)	0.366

Abbreviations: BMI: body mass index; FSH: follicle-stimulating hormone; LH: luteinizing hormone; E2: estradiol; T: testosterone; AMH: anti-Müllerian hormone; AFC: antral follicle count; rFSH: recombinant follicle-stimulating hormone. * Utilizable embryos on Day 3 were identified according to morphological criteria, including normally fertilized embryos with four blastomeres and fragmentation < 10%, as well as embryos with five or more blastomeres and fragmentation < 25% on Day 3. ^a^: The Mann–Whitney U test was used for continuous variables and the Chi-square test for categorical variables. A *p* value < 0.05 was considered statistically significant.

**Table 2:** Univariate and multivariate linear regression analysis of factors associated with average daily rFSH dose.

Variables	Univariate Analysis		Multivariate Analysis		
	B (95% CIs)	*p*	B (95% CIs)	*p*	Adjusted *R*^2^
Female age (years)	4.162 (3.020~5.304)	<0.001	3.120 (2.131~4.110)	<0.001	0.404
BMI (kg/m^2^)	4.798 (3.431~6.164)	<0.001	4.260 (3.078~5.443)	<0.001	
Basal FSH (mIU/mL)	4.972 (1.772~8.172)	0.002	6.790 (3.939~9.640)	<0.001	
Basal LH (mIU/mL)	−1.892(−2.980~−0.804)	0.001	−1.806 (−2.808~−0.805)	<0.001	
AMH (ng/mL)	−2.144 (−3.130~−1.158)	<0.001	−0.881(−1.756~−0.005)	0.049	
AFC (n)	−1.155 (−1.733~−0.577)	<0.001	−0.7006 (−1.210~−0.202)	0.006	

Abbreviations: BMI: body mass index; FSH: follicle-stimulating hormone; LH: luteinizing hormone; AMH: anti-Müllerian hormone; AFC: antral follicle count.

**Table 3:** Validation of the average daily rFSH dose prediction algorithm.

Group	Paired *t*-Test *p*	*ρ*	MAE (IU)	RMSE (IU)	Deviation ≤ 20%
Modeling group (*n* = 258)	0.986	0.646	21.82	29.75	77.91%
Validation group (*n* = 111)	0.540	0.676	23.87	31.89	67.57%

Abbreviations: rFSH: recombinant follicle-stimulating hormone; ρ: Spearman’s correlation coefficient; MAE: mean absolute error; RMSE: root mean square error.

## Data Availability

Data can be accessed by contacting the corresponding authors.
